# Angry and Fearful Face Conflict Effects in Post-traumatic Stress Disorder

**DOI:** 10.3389/fpsyg.2019.00136

**Published:** 2019-02-05

**Authors:** Victoria Ashley, Diane Swick

**Affiliations:** ^1^Research Service, Veterans Affairs Northern California Health Care System, Martinez, CA, United States; ^2^Department of Neurology, University of California, Davis, Davis, CA, United States

**Keywords:** emotion, PTSD, flanker, faces, anger, attention bias

## Abstract

In the presence of threatening stimuli, post-traumatic stress disorder (PTSD) can manifest as hypervigilance for threat and disrupted attentional control. PTSD patients have shown exaggerated interference effects on tasks using trauma-related or threat stimuli. In studies of PTSD, faces with negative expressions are often used as threat stimuli, yet angry and fearful facial expressions may elicit different responses. The modified Eriksen flanker task, or the emotional face flanker, has been used to examine response interference. We compared 23 PTSD patients and 23 military controls on an emotional face flanker task using angry, fearful and neutral expressions. Participants identified the emotion of a central target face flanked by faces with either congruent or incongruent emotions. As expected, both groups showed slower reaction times (RTs) and decreased accuracy on emotional target faces, relative to neutral. Unexpectedly, both groups showed nearly identical interference effects on fearful and neutral target trials. However, *post hoc* testing suggested that PTSD patients showed faster RTs than controls on congruent angry faces (target and flanker faces both angry) relative to incongruent, although this finding should be interpreted with caution. This possible RT facilitation effect with angry, but not fearful faces, also correlated positively with self-report measures of PTSD symptoms. These results suggest that PTSD patients may be more vigilant for, or primed to respond to, the appearance of angry faces, relative to fearful, but further study is needed.

## Introduction

Post-traumatic stress disorder (PTSD) involves a constellation of symptoms following trauma exposure, such as re-experiencing of the trauma, hypervigilance for threat and avoidance of trauma reminders (DSM-5, American Psychiatric Association, 2013). Studies of PTSD patients have found cognitive deficits in inhibitory control (Leskin and White, 2007; Pineles et al., 2007; Swick et al., 2012; DeGutis et al., 2015) and altered reactivity to emotional stimuli, both trauma-specific (Ashley et al., 2013; Todd et al., 2015) and general threat stimuli, including negative facial expressions, i.e., anger, fear, sadness, and disgust (Hariri et al., 2002; Kirsch et al., 2005; Shin et al., 2005; Phan et al., 2008; MacNamera et al., 2013; Schönenberg and Abdelrahman, 2013; Dunkley et al., 2016; DiGangi et al., 2017a; Heesink et al., 2017; Rubin et al., 2017; Zinchenko et al., 2017). Although the facial expressions of anger and fear are often used interchangeably as ‘threat’ stimuli, anger and fear may convey different types of threat and elicit different responses (Davis et al., 2011; Berggren and Derakshan, 2013; Taylor and Whalen, 2014; Woodward et al., 2017). While fear communicates danger in the surroundings (externally oriented for environmental monitoring), anger conveys a more proximal, central and direct threat, focusing attention inward (Davis et al., 2011; Berggren and Derakshan, 2013; Taylor and Whalen, 2014).

Studies comparing behavioral and neural responses to angry and fearful facial expressions in healthy adults have found varying results (Engen et al., 2017). For example, in an eye-tracking task of initial orienting with high- and low-anxious undergraduates, Mogg et al. (2007) found similar patterns of attentional biases for both anger and fearful face expressions. But Ewbank et al. (2009), using a same/different matching task with faces and houses, reported selectively different amygdala responses to fearful and angry facial expressions, depending on attention and anxiety, with attended expressions associated with a larger right amygdala response to angry compared to fearful, whereas unattended expressions were associated with a larger left amygdala response to fearful faces. In a forced-choice emotion recognition task with blurred faces directed toward or away from an observer, Hortensius et al. (2016) found that anger was better recognized when an expression was directed toward an observer, yet fear was better recognized when directed away from an observer, underscoring the potentially different ecological roles of these expressions.

Theories of PTSD suggest that fear learning becomes dysregulated and hyper-responsive, leading to symptoms such as hypervigilance for threat and trauma avoidance (Wilker and Kolassa, 2013). Historically, studies of emotional dysregulation in PTSD have used fearful expressions, which can reliably activate the amygdala, a structure relevant for its role in fear extinction. For example, several imaging studies using passive viewing tasks of fearful and happy face expressions (masked and unmasked) with PTSD patients have reported amygdala hyper-responsivity to fear expressions, relative to happy, together with attenuated “top-down” responsivity in medial frontal cortex (Rauch et al., 2000; Armony et al., 2005; Shin et al., 2005). None of these studies used angry faces. But more than other anxiety disorders, PTSD has also been associated with anger (Chemtob et al., 1994; Jakupcak et al., 2007; Elbogen et al., 2010; Olatunji et al., 2010). Anger and aggression problems in veterans with PTSD are associated with increased threat reactivity, an inability to regulate arousal, and a lowered threshold for perceiving threat stimuli, compared to control veterans (Heesink et al., 2017). Studies of PTSD using both angry and fearful expression faces have found mixed results, such as increased activations of the amygdala for fearful but not angry faces (Simmons et al., 2011), larger early event-related potential components to angry (P1 and P2 amplitudes) and fear (P2) faces in patients with high symptom levels (Zuj et al., 2017), and slower response times and worse performance for angry relative to fearful and happy expression faces (DiGangi et al., 2017b).

In an earlier pilot experiment, we used a simple face identification task with PTSD patients and military controls to measure error rates and expression misattributions (happy, angry, fearful, sad, surprised, or neutral) (Ashley et al., 2012). Although no significant group differences were found, some interesting trends emerged in patients’ responses to angry and fearful expression faces: patients, but not controls, tended to be more accurate on angry faces and less on fearful, more likely to misattribute anger to other expressions, and tended to show a positive correlation between accuracy on angry faces and self-reported symptoms of PTSD and depression. These preliminary results, along with other findings in the literature (DiGangi et al., 2017b; Heesink et al., 2017), prompted us to look more closely at the relationship between these two negative expressions in PTSD patients. We used a more difficult decision-making task that involved conflict between target and distractors to look at possible facilitation and interference effects.

The current study examined the role of vigilance for threat stimuli in PTSD when these stimuli are both task relevant and irrelevant. We used a modified Eriksen flanker task (Eriksen and Eriksen, 1974) in which participants must identify the expression of a central target face (relevant) while ignoring two flanker faces (irrelevant) (Moser et al., 2008; Chen et al., 2016). Based on previous research comparing threatening and neutral non-threatening faces (Phan et al., 2008; DiGangi et al., 2017a), we expected to see significantly slower RTs for angry and fearful targets in both groups, but to a greater extent in PTSD patients. However, those studies used an emotion matching task, while our experiment required explicit identification of emotions. Anger has been increasingly recognized as an issue in combat veterans with PTSD (Heesink et al., 2017, 2018; Forbes et al., 2018). Therefore, we predicted that angry target faces would be more disruptive (leading to slower RTs) than fearful or neutral targets. For flanker expressions, we expected slower RTs in the patients when neutral targets were paired with angry or fearful flankers, relative to neutral targets paired with neutral flankers (Zinchenko et al., 2017). We also predicted that such interference effects would correlate positively with scores on the PTSD checklist (PCL), Beck Depression Inventory (BDI), and Anger Questionnaire (AQ). Additionally, we expected higher accuracy scores for PTSD patients relative to controls on angry faces, based on the findings of Anaki et al. (2012) in combat vs. non-combat veterans, which is in accord with our pilot study.

## Materials and Methods

### Participants

Participants were recruited from clinics at the Veterans Affairs of Northern California Health Care System, fliers placed in local military offices, and internet postings. Thirty-one OEF/OIF war veterans with PTSD (PTSDs; 2 female) and 29 military controls (MCs; 3 female) enrolled in the study. Eight PTSD patients and 5 military controls were removed due to performance issues (see section “Data Analysis”) and one MC was removed based on exclusion criteria that were not revealed at the initial interview. Thus, the reported results include 23 PTSD patients (1 female, mean age: 34.2 years, *SD*: 7.1) and 23 military controls (3 females; mean age: 38.8 years, *SD*: 9.0). See Table 1 for details on demographic data.

**Table 1:** Demographic information and symptom severity scores for participants with PTSD and military controls.

	PTSD patients (*n* = 23)	Military controls (*n* = 23)
Age (years)	34.2 ± 7.1 (24–50)	38.8 ± 9.0 (26–54)
Education (years)	14.1 ± 1.3^∗∗^ (12–16)	15.2 ± 2.1 (12–19)
Handedness	21 R, 1 L, 1 amb	16 R, 7 L
Combat-exposed	23	11
PCL-5	45.0 ± 14.8^∗∗∗^	9.2 ± 7.5
• Intrusion	11.0 (4.35)^∗∗∗^	2.3 (2.2)
• Avoidance	4.6 (2.5)^∗∗∗^	1.2 (1.6)
• Neg Alterations	14.0 (6.9)^∗∗∗^	2.4 (2.7)
• Increased arousal	15.4 (5.2)^∗∗∗^	3.3 (3.4)
BDI	19.4 ± 10.8^∗∗∗^	5.5 ± 11.4
AQ	91.3 ± 22.4^∗∗∗^	57.5 ± 11.6
CFQ	59.5 ± 14.0^∗∗∗^	19.6 ± 11.6
CES	23.7 ± 7.1^∗∗∗^	5.4 ± 6.8
RTs (ms)	752.1 ± 90.6	752.9 ± 110.4

*The means (standard deviations) are given for age, education, self-report measures and overall RTs. ^∗∗^ significantly different from controls at p < 0.01; ^∗∗∗^ significantly different from controls at p < 0.005. R, right; L, left; amb, ambidextrous; PCL-5, PTSD checklist for DSM-5; BDI, Beck Depression Inventory; AQ, Aggression Questionnaire; CFQ, Cognitive Failures Questionnaire; CES, Combat Exposure Scale.*

Exclusion criteria included any neurological disorder other than mild traumatic brain injury (TBI) (e.g., epilepsy), a severe psychiatric disorder (i.e., schizophrenia, bipolar disorder), having PTSD not due to OEF/OIF events, having a childhood TBI or a moderate to severe TBI. Common mental health comorbidities in PTSD (e.g., depression, generalized anxiety) were allowed. A history of mild TBI was also allowed, since combat-related PTSD patients also often have mTBI(s) due to combat incidents (Carlson et al., 2011). Other exclusionary conditions for controls included a history of TBI or PTSD and current depression or anxiety.

### Clinical Interview and Diagnosis

The diagnosis of PTSD was made through a semi-structured clinical interview by VA mental health providers using DSM-IV criteria or the Clinician-Administered PTSD Scale (CAPS). Mild TBI was diagnosed by a neurologist based on a semi-structured clinical interview and patient self-report of the following criteria from the VA/DoD Clinical Practice Guidelines: loss of consciousness (LOC) < 30 min or altered mental status (e.g., feeling dazed or disoriented) with post-traumatic amnesia <24 h (The Management of Concussion/Mild TBI Working Group, 2009). Twelve of the 23 PTSD patients reported or were diagnosed with a mTBI, typically due to IED blast exposure. Diagnosis of mTBI and PTSD in patients enrolled in our study was confirmed via a review of the VA’s Computerized Patient Record System (CPRS) and other available VA medical records to the fullest extent possible.

The Institutional Review Board of the VA Northern California Health Care System approved the experimental protocol. All participants gave informed consent prior to starting the experiment and were paid $20/hour and transportation costs for their participation. The research was conducted in accordance with the Declaration of Helsinki.

### Stimuli

**FIGURE 1:**
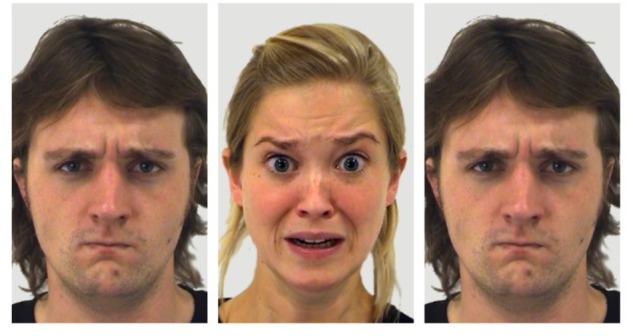
Face flanker stimulus example showing incongruent fear (F–A). The central target face shows a fearful expression and the two flanker faces show an angry expression. These faces are from the Amsterdam Dynamic Facial Expression Set (ADFES) (Van Der Schalk et al., 2011), with the central face being identified as F03AFS, and the two flanker faces as M04ANS. Written informed consent from the depicted individuals, and permission from the copyright holders for the publication of these images, was provided by the University of Amsterdam Department of Psychology.

Each stimulus array consisted of 3 faces in a row, located in the center of the screen on a light gray background: one central face (target) and two identical distractor faces on either side (flankers) (Figure 1). To avoid the repetition of small numbers of emotional stimuli, which can dilute results with habituation or priming effects, we created 192 unique target-flanker face sets, made up of 64 individuals (35 males and 29 females) each displaying angry, fearful or neutral expressions. Half of all trials were congruent arrays, and half incongruent. Congruent face array conditions included angry–angry (A–A), fear–fear (F–F) and neutral–neutral (N–N), where, for example, “A–A” denotes an angry target with angry flankers. Incongruent face array conditions included A–F, A–N, F–A, F–N, N–A, N–F, where, for example, “A–F” denotes an angry target with fearful flankers.

Because we sought to examine the role of realistic ecologically valid stimuli (Wheatley et al., 2011) on behavioral reaction times and accuracy between two groups of combat veterans, we used an ethnically diverse set of color faces without masking (Liu et al., 2013; Dickter et al., 2018). Faces were used from four face sets: the Amsterdam Dynamic Facial Expression Set (ADFES) (Van Der Schalk et al., 2011), the Karolinska Directed Emotional Faces (KDEF) (Lundqvist et al., 1998), the Warsaw Set of Emotional Facial Expression Pictures (WSEFEP) (Olszanowski et al., 2015) and the Radboud Faces Database (RFD) (Langner et al., 2010). Ethnicities included North-European and Mediterranean/Moroccan Dutch. Faces were cropped, color-adjusted, sized and aligned by eyes as necessary for uniformity using Adobe Photoshop and Adobe Illustrator. ImageMagick was used to create the 3-face arrays. A thin white rectangular frame surrounded each face, measuring 6.5 cm in width and 10.69 cm in height (184 pixels × 303 pixels), with the full 3-face set in the frame measuring and 20.78 cm in width and 11.28 cm in height (589 pixels × 320 pixels).

### Procedure

Participants viewed 6 blocks of face stimuli in pseudo randomized order on a computer screen in a dimly lit and sound attenuated room, seated at about 70 cm from the screen. Each block was composed of 72 trials, half congruent and half incongruent, with an equal number (24) of angry, fearful and neutral target face expressions per block, for a total of 432 trials. Trials started with a fixation cross for 400 ms, followed by the 2 flanker faces for 200 ms. Then a target face appeared and was displayed together with the flankers for 700 ms, followed by a 900 ms blank screen, for a total trial duration of 2200 ms. Participants were instructed to press one of three keys indicating the correct emotion of the central target face (angry, fearful, or neutral) while ignoring the flanker faces. They began with a short practice block. Key order was counterbalanced across subjects.

### Self-Report Measures

Five self-report measures were administered following the behavioral task: the Beck Depression Inventory (BDI) (Beck et al., 1961), a commonly used 21-item assessment of levels of depression in the past few days; the PTSD Checklist 5 (PCL-5) (Weathers et al., 2013), a 20-item measure which asks about levels of PTSD symptoms due to “stressful military experiences” that the subject has been bothered by in the past month; the Aggression Questionnaire (AQ) (Buss and Warren, 2000), a 34-item series of phrases encompassing different levels of anger and aggression, such as, “My friends say that I argue a lot,” with a 5 level range of responses ranging from, “not at all like me” to “completely like me”; the Cognitive Failures Questionnaire (CFQ) (Broadbent et al., 1982), a 25-item measure of self-reported failures in memory, perception, and motor function on everyday tasks in the past 6 months, with 5 levels of responses ranging from “Very Often” to “Never”; and the Combat Exposure Scale (CES) (Keane et al., 1989), a 7-item self-report measure to assess how many combat stressors a participant was exposed to during deployment, with questions like, “Were you ever surrounded by the enemy?”, with a 5 level range of quantitative responses, such as “No” to “51+times”.

### Data Analysis

Data were trimmed to remove premature RTs (<300 ms; PTSD: 0.9%; MC 0.0%) (Ratcliff, 1993; Yu et al., 2018). Only correct responses were included in the RT analyses (percentage of erroneous responses removed: PTSDs = 14.4%; MCs = 13.1%). Eight PTSD patients and 5 military controls were removed due to performance issues, in keeping with previous behavioral exclusion criteria (Ratcliff, 1993; Wurm et al., 2004; Ashley and Swick, 2009; Ashley et al., 2013; Yu et al., 2018). Exclusion criteria included making more than 25% decision errors (6 PTSDs; 5 MCs), making more than 25% decision errors plus missed responses (1 PTSD), and mean response RTs that fell more than 3 SDs outside the mean of the group (1 PTSD with an average RT of 1161 ms; PTSD group mean: 752 ms). Thus, the reported results include a total of 23 PTSD patients and 23 military controls. Importantly, the number of patients and controls removed for excessive emotion decision errors did not differ. These errors were primarily confusions between fear and anger.

Although groups were matched on age [*t*(44) = −1.66, *p* = 0.11], controls had more years of education [PTSDs: *M* = 14.2, *SD* = 1.4; MCs: *M* = 15.7, *SD* = 2.2; *t*(44) = 2.8, *p* < 0.007, Cohen’s *d* = 0.827]. This is often due to the inability of veterans with PTSD to return to school following their military service (e.g., Mac Donald et al., 2017).

Reaction time and error results were analyzed in a 2 × 3 × 3 repeated measures ANOVA with Target Valence (angry, fearful, and neutral) and Flanker Valence (angry, fearful, and neutral) as the within-subject factors, and Group (PTSDs, MCs) as the between-subjects factor. The Greenhouse–Geisser procedure was used to correct for any violations of sphericity and Bonferroni corrected α of 0.005 was applied to *post hoc t*-tests. Spearman correlations between self-reported symptom scores with RTs and accuracy scores, used a corrected α of 0.005. JASP statistical software (version 0.8.1.1) was also used to calculate statistical data, including Bayes factors. The raw data supporting the conclusions of this manuscript will be made available by the authors, without undue reservation, to any qualified researcher.

## Results

### Reaction Times

Reaction time results of the repeated measures ANOVA indicated no main effect of Group [*F*(1,44) = 0.001, *p* = 0.977, $\eta_{p}^{2}$ = 0.0], with nearly identical mean group RTs (PTSDs = 752.1 ms; MCs = 752.9 ms). A significant main effect was shown for Target Valence [*F*(2,88) = 60.1, *p* < 0.0001, $\eta_{p}^{2}$ = 0.58], with paired-*t*-test comparisons revealing that both groups were slower on emotional target faces, relative to neutral [*t*(45) = 9.81, *p* < 0.001, *d* = 1.45]. Of the emotional target faces, participants were slower on fearful relative to angry expressions (*p* < 0.02, mean diff: PTSDs = 28.5 ms; MCs = 19.3 ms). The main effect of Flanker Valence was not significant [*F*(2,88) = 2.46, *p* = 0.09, $\eta_{p}^{2}$ = 0.05], indicating that overall, flanker valence did not influence target RTs. This finding is not that unusual for face flanker studies, some of which do not find behavioral effects (e.g., Moser et al., 2008; Chen et al., 2016). However, a significant interaction was found for Target Valence × Flanker Valence, [*F*(4,4) = 8.5, *p* < 0.0001, $\eta_{p}^{2}$ = 0.16], suggesting that flanker valence did affect target RTs in a more specific way. No interaction effects were shown for Flanker Valence × Group (*p* > 0.1), Target Valence × Group (*p* > 0.6), or Target Valence × Flanker Valence × Group (*p* > 0.3). For mean RTs and accuracy, see Table 2.

**Table 2:** Mean reaction times and percent accuracy are shown for PTSD patients and military controls.

	Targets		
	Angry	Fear	Neutral
**RT (ms)**			
PTSD	759.03 (86.7)	787.49 (93.6)	709.77 (73.75)
Controls	767.58 (107.4)	786.84 (111.6)	704.21 (95.6)
**Accuracy (%)**			
PTSD	81.84 (8.2)	82.19 (8.1)	92.85 (5.2)
Controls	82.19 (8.6)	84.84 (7.3)	94.03 (4.5)

*Responses are collapsed across the 3 flanker valences. The means (standard deviations) are in milliseconds for RT (reaction time) and in percentages for error rate.*

**FIGURE 2:**
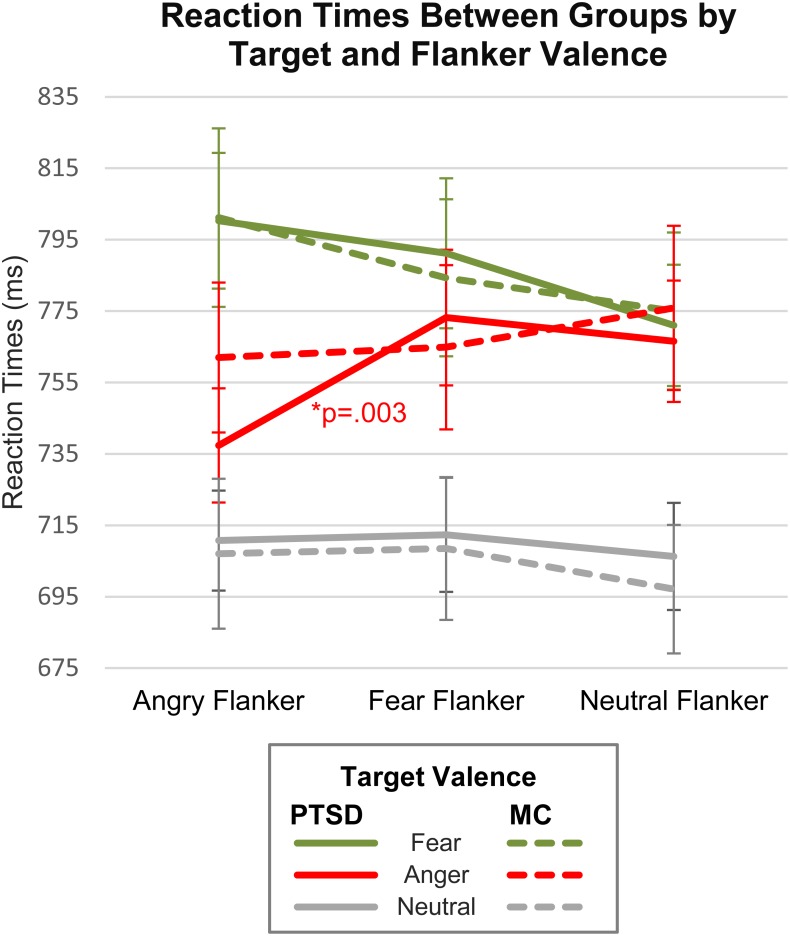
Reaction times by valence, congruency and group. Congruent conditions include A–A, F–F, and N–N. Incongruent conditions include A–F, A–N, F–A, F–N, N–A, and N–F.

While no significant omnibus interaction effect (Group × Target × Flanker) was found, we felt that separately analyzing target valences using a corrected Bonferroni α of 0.005 and analyses using Bayes factors, could more closely quantify the strength of evidence for any possible group differences. In addition, some face studies have noted the need to address the role of valence-specific effects, particularly with threat faces, since negative valence expressions can override other factors of interest, such as congruency, gender, identity, etc. (Weinberg et al., 2012; Schulte Holthausen et al., 2016). Thus, we performed repeated measures ANOVAs for each Target Valence separately and found main effects of Flanker Valence for angry targets [*F*(2,88) = 8.58, *p* < 0.001, $\eta_{p}^{2}$ = 0.151] and fearful targets [*F*(2,88) = 8.02, *p* = 0.001, $\eta_{p}^{2}$ = 0.153], but not neutral targets [*F*(2,88) = 1.75, *p* = 0.18, $\eta_{p}^{2}$ = 0.038]. A Flanker Valence × Group interaction was observed for angry targets only [*F*(2,88) = 4.15, *p* = 0.020, $\eta_{p}^{2}$ = 0.073]. Bonferroni corrected *post hoc* comparisons within each group indicated that PTSD patients, but not military controls, were significantly faster on congruent angry faces (A–A) relative to incongruent [PTSDs: (A–A vs. A–F): *t*(22) = −4.24, *p* < 0.001, *d* = 0.884; (A–A vs. A–N): *t*(22) = −3.08, *p* = 0.005, *d* = 0.643; MCs: (A–A vs. A–F): *t*(22) = −0.49, *p* = 0.623, *d* = 0.102; (A–A vs. A–N): *t*(22) = −1.88, *p* = 0.072] (Table 3 and Figure 2). Interference and facilitation effects for all conditions, measured as incongruent minus congruent RTs, are shown in Table 4.

**Table 3:** Mean reaction times (and SDs) for congruent and incongruent targets for PTSD patients and military controls.

RT (ms)	Congruent			Incongruent			
	Angry (A–A)	Fear (F–F)	Neutral (N–N)	Angry (A–F)	Fear (F–A)	Neutral (N–A)	Neutral (N–F)
PTSD (*n* = 23)	737.37 (77.7)	791.19 (104.8)	706.27 (75.0)	773.18 (95.7)	800.30 (94.2)	710.71 (69.3)	712.34 (79.8)
Controls (*n* = 23)	761.99 (102.3)	784.33 (108.3)	697.11 (88.0)	764.86 (111.6)	801.19 (121.3)	707.03 (103.8)	708.49 (98.2)

*Congruent conditions are A–A, F–F, and N–N; incongruent conditions are A–F, F–A, N–A, and N–F. The means (standard deviations) are in milliseconds.*

**Table 4:** Reaction time congruency effects.

	A–F -A–A	A–N - A–A	F–A - F–F	F–N - F–F	N–A - N–N	N–F - N–N
PTSD (*n* = 23)	35.82 (40.53)	29.18 (45.39)	9.11 (67.96)	20.20 (51.63)	4.44 (35.46)	6.07 (32.97)
Controls (*n* = 23)	2.87 (28.09)	13.89 (35.29)	16.85 (38.02)	9.32 (32.49)	9.92 (37.01)	11.39 (33.28)

*Mean reaction time differences for incongruent and congruent conditions. The means (standard deviations) are in milliseconds.*

To more closely quantify the strength of evidence for any possible group differences, Bayes Factors (BF10) were calculated using JASP statistical software version 0.8.1.1 (JASP Team, 2017). BF10 < 1 provides evidence in favor of the null hypothesis (H0), while BF10 > 1 favors the alternate hypothesis (H1). We conducted JZS Bayes Factor repeated measures ANOVAs (Morey et al., 2015) with default prior scales for each Target Valence separately.

For angry targets, the Flanker Valence × Group interaction model was preferred (BF10 = 68.98) to the null model. Overall, this can be considered “very strong” evidence (Wagenmakers et al., 2017) in favor of the Flanker Valence × Group interaction model. However, the main effects model of Flanker Valence (BF10 = 26.55) was also preferred to the null model. Overall, the interaction model was preferred to the main effects model (68.98/26.55 = 2.598), but only weakly. This suggests the following results should be interpreted with caution. Bayesian between group independent samples *t*-tests were then conducted for flanker interference effects (incongruent minus congruent RTs) on angry targets. A–F interference differences between groups (BF10 = 14.61) were 14 times more likely than the null, which is considered “strong” evidence.

For fearful targets, the main effects model with Flanker Valence was preferred (BF10 = 45.56, “very strong”) over the null model. The Flanker Valence × Group interaction model was moderately preferred over the null (BF10 = 4.39). Hence, the main effect of flanker valence for fearful targets was preferred to the interaction model that included group (4.39/45.56 = 0.096). Bayesian paired sample *t*-tests indicated that both groups were slower on fearful targets with angry flankers (F–A) relative to neutral (F–N) (PTSDs: BF10 = 6.30, MCs: BF10 = 24.42). For neutral targets, the null was more likely than both the main effects model of Flanker Valence (BF10 = 0.313), and the Flanker Valence × Group interaction model (BF10 = 0.026, “very strong” evidence against the interaction).

Finally, we conducted an analysis to determine if slowing on negative emotional target trials was carrying over onto neutral target trials by comparing RTs on neutral target trials following angry, fearful or neutral target trials. No significant effects were found (*p* > 0.2), with both groups slower on angry and fearful flanker conditions by just 5–12 ms.

### Accuracy

A repeated measures ANOVA conducted for accuracy scores showed no significant main effect of Group [*F*(1,44) = 1.12, *p* = 0.296, $\eta_{p}^{2}$ = 0.025], nor any Group interaction effects (*p* > 0.42), indicating that both groups performed similarly, overall, on the task. Main effects of Target Valence [*F*(2,88) = 58.01, *p* < 0.001, $\eta_{p}^{2}$ = 0.566], Flanker Valence [*F*(2,88) = 5.32, *p* = 0.007, $\eta_{p}^{2}$ = 0.107], and Target Valence × Flanker Valence were shown [*F*(4,176) = 3.29, *p* = 0.016, $\eta_{p}^{2}$ = 0.068], with the largest effect being higher accuracy on Neutral targets (93.4%) relative to angry (82.0%) or fearful targets (83.5%). *Post hoc* comparisons of flanker valence accuracy indicated that across groups, angry flankers elicited higher accuracy (87.23%) than fearful flankers (85.67%) [*t*(88) = 3.21, *p* = 0.005]. To follow up on the Target Valence × Flanker Valence interaction, accuracy for angry targets was examined in a separate repeated measures ANOVA. This indicated a significant main effect of Flanker Valence [*F*(2,88) = 8.79, *p* < 0.001, $\eta_{p}^{2}$ = 0.164], with Bonferroni corrected *post hoc* comparisons showing the highest accuracy for congruent angry faces (A–A: 84.25%) relative to incongruent (A–F: 81.08%, A–N: 80.71%) [A–A, A–F: *t*(2,88) = 3.17, *p* = 0.007; A–A, A–N: *t*(2,88) = 4.21, *p* < 0.001]. No accuracy differences were shown on fearful or neutral target faces (*p* > 0.3).

For PTSD patients, but not military controls, better accuracy was associated with faster RTs on angry target faces [*r*(1,67) = 0.37, *p* = 0.002], but not on fearful [*r*(1,67) = 0.25, *p* = 0.04] or neutral (*p* > 0.15) target faces.

### Self-Report Questionnaires

**FIGURE 3:**
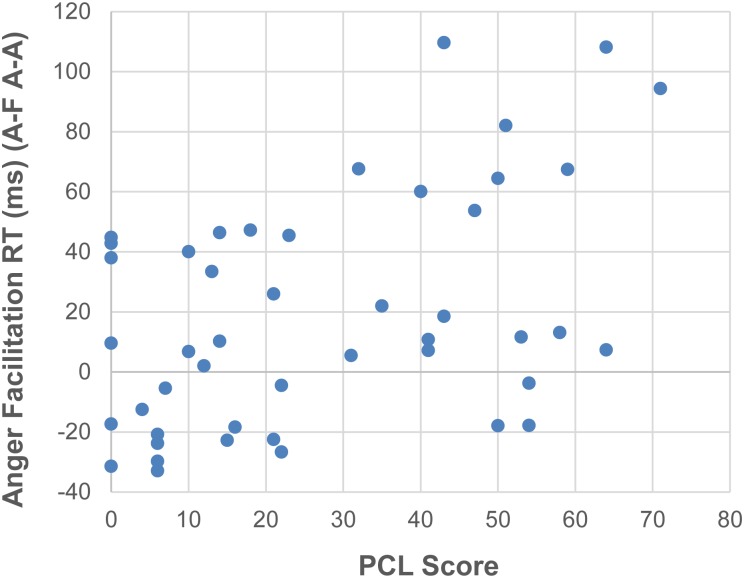
Positive correlation between facilitation RTs on angry target faces (A–F – A–A) and PTSD symptoms on the PCL-5.

As expected, PTSD patients showed significantly higher scores than MCs on the PCL [*t*(44) = 10.33, *p* < 0.001, *d* = 3.05], BDI [*t*(44) = 5.9, *p* < 0.001, *d* = 1.74], AQ [*t*(44) = 6.44, *p* < 0.001, *d* = 1.40], CFQ [*t*(44) = 10.52, *p* < 0.001, *d* = 3.10] and CES [*t*(44) = 8.17, *p* < 0.001, *d* = 2.41] (for details, see Table 1). Since our previous study with PTSD patients found significant relationships between self-report questionnaire scores of PTSD symptoms and reaction time interference from trauma-related words (Ashley et al., 2013), we wondered if the face flanker facilitation effects we observed could be similarly related to PTSD symptoms. Using *post hoc* Spearman correlations between RT facilitation for angry target faces (A–F minus A–A) and self-report questionnaire scores (PCL-5, BDI, CES, CFQ, and AQ), we found that across all participants, RT facilitation on angry target faces correlated positively with PTSD symptoms on the PCL-5 [*rho* = 0.414, *p* = 0.004] (Figure 3). The rest of the self-report results did not survive correction: depression scores on the BDI [*rho* = 0.399, *p* = 0.006], levels of combat exposure on the CES [*rho* = 0.346, *p* = 0.018], cognitive failure scores on the CFQ [*rho* = 0.333, *p* = 0.024] or aggression scores on the AQ [*rho* = 0.264, *p* = 0.076]. Too few of our PTSD patients scored low enough on the AQ to conduct a meaningful comparison between those with low and high AQ scores. For example, while most of our military controls scored in the 40’s to 60’s on the AQ (mean of 57.5, SD 11.6), only four of our PTSD patients scored in the 50’s and 60’s (mean of 91.3, *SD* 22.4).

## Education

To test whether the lower education in the PTSD group affected the findings, we compared a subset (*n* = 12) from each group which were not statistically different on education (PTs: *M* = 15.04, *SD* = 1.3; MCs: *M* = 14.2, *SD* = 1.3) [*t*(22) = 1.61, *p* = 0.121, *d* = 0.658] and found that the overall effects were still significantly different on Target Valence [*F*(2,44) = 21.26, *p* < 0.0001, $\eta_{p}^{2}$ = 0.491], Target Valence × Flanker Valence [*F*(4,88) = 5.87, *p* < 0.001, $\eta_{p}^{2}$ = 0.200], and that the interaction for Target Valence × Group for angry faces still existed [*F*(2,44) = 3.89, *p* = 0.03, $\eta_{p}^{2}$ = 0.125]. Additionally, both groups were significantly more accurate on neutral target faces than anger or fear [*F*(2,44) = 34.46, *p* < 0.001, $\eta_{p}^{2}$ = 0.608] and accuracy did not differ between groups [*F*(1,22) = 0.588, *p* = 0.451, $\eta_{p}^{2}$ = 0.026], with no group interaction indicated [*F*(2,44) = 0.200, *p* = 0.82, $\eta_{p}^{2}$ = 0.004].

## Discussion

The purpose of this study was to examine the roles of two facial expressions of threat – anger and fear – on the ability of PTSD patients and controls to identify a central target face expression while ignoring two task-irrelevant flanking faces. The results broadly support a similarity between the groups in their ability to promptly and accurately identify target emotional facial expressions, despite the presence of adjacent distracting flanker expressions. Both groups showed an emotional slowing effect (slower to respond to emotional target expressions relative to neutral) and were slowest on fearful targets relative to angry or neutral. However, a key unexpected difference between groups was observed: PTSD patients, but not controls, were significantly faster to identify the target expression of congruent angry face arrays (A–A), relative to incongruent (A–F specifically), despite groups performing the same on nearly all other combinations of expressions of angry, fearful and neutral. This facilitation effect also correlated positively across groups with self-reported PTSD symptoms (PCL).

While faster RTs could be related to impulsivity, PTSD patients did not show a decrement in accuracy with facilitated response times to congruent angry faces. Rather, both groups were more accurate on angry flankers relative to fearful flankers, and were more accurate on congruent (A–A) relative to incongruent (A–N, A–F). That PTSD patients were both more accurate and faster on congruent angry faces arrays suggests they may have been more vigilant for, or primed to respond to these faces.

Our prediction that PTSD patients would show larger emotional slowing effects on angry and fear target faces relative to military controls was not obtained. We based this prediction on studies that found slower RTs for angry faces relative to fearful and happy, in both controls and PTSD patients (MacNamera et al., 2013; DiGangi et al., 2017b). However, these studies used an emotional face matching task, which differs in several respects from our flanker task. In the face matching task, 3 faces were presented for 3000 ms in a triangular arrangement, with one face centered in the top-half of the screen and two faces spaced apart in the bottom-half of the screen. Participants were to select one of the two faces at the bottom of the screen that had the same emotional expression as the face centered in the top portion of the screen. In our task, faces were only seen together for a total of 700 ms, and the central face was always the target. Additionally, the face matching task did not involve an explicit emotion identification and did not include a neutral expression.

We also did not predict that the patients would be faster on congruent angry target faces than MCs, so this facilitation effect deserves further study. Some studies of individuals with high trait anger have found RT facilitation effects (Veenstra et al., 2017) and enhanced detection of masked facial expressions of anger in high trait anxiety individuals (Damjanovic et al., 2017). Among veterans with PTSD, level of executive functioning and PTSD symptoms have been found to correlate with reactivity to angry faces (Dunkley et al., 2016; DiGangi et al., 2017b). Termed ‘the anger superiority effect’, studies show that, in many cases, angry faces, more than other negative expressions, are more rapidly detected among arrays of facial expressions and may elicit improved visual short-term memory, independent of arousal, emotional intensity or task relevance (Jackson et al., 2009; Maran et al., 2015; Lo and Cheng, 2017).

We were surprised to not find a significant flanker interference effect for neutral target faces (slower on N–A and N–F relative to N–N). Was this lack of a conflict effect due to the design? In other words, if only neutral target faces been used (with a task of gender identification), would emotional flankers then have been more distracting? One emotional face flanker study in controls used a gender identification task (Kim et al., 2017). Significantly larger congruency effects were found when target faces were neutral and flanker faces were emotional, compared to when target faces were emotional and flanker faces were neutral (Kim et al., 2017). In the current mixed block design, emotional targets appeared to play a key role in the impact of flanker expressions. Additionally, given the high accuracy and faster RTs on neutral target faces, it is possible that the ease in identifying neutral expressions on target faces overshadowed any potential flanker interference effects.

Identifying stimuli that can index changes in emotional responses in PTSD patients may contribute to therapies and measures designed to assess potential improvements in everyday function. Recent studies of potential therapies for PTSD have found distinctions between angry and fearful face expressions. In an fMRI pilot study of the outcome of a mindfulness exposure therapy for veterans with PTSD, King et al. (2016) found pre- to -post therapy improvements in PTSD symptoms correlated with increased responses to angry faces in left amygdala (among other areas), but not fearful faces. And in an eye-tracking study of visual attention to negative and neutral face pairs in veterans with PTSD, Woodward et al. (2017), found that the presence of a familiar service canine specifically reduced attention toward angry faces, but not fearful or happy faces, compared to viewing the faces without the canine.

While the interpretive limitations of our study preclude practical applications for diagnosis and treatment outcomes, an increasing number of population studies in veterans have revealed that anger increases suicide risk (McKinney et al., 2017; Wilks et al., 2018). Given the higher rate of gun ownership in veterans, firearm deaths by suicide are associated with PTSD, substance abuse, and social disconnectedness (Desai et al., 2008). Anger may play a role in a diminished level of social capital, defined as ‘the level of community organizational life, engagement in public affairs, community volunteerism, informal sociability, and social trust’ (Desai et al., 2008).

Several limitations of our study should be noted. First, some participants showed a high error rate that resulted in the removal of their scores from the study. However, the number of participants removed was similar for each group (7 PTSDs; 6 MCs) and we used a consistent guideline for removal (those with a more than 25% error rate). Another limitation was that PTSD patients had fewer years of education than military controls. However, subgroup analyses did not show differences for the major dependent measures, suggesting that education was not likely responsible for group differences. Additionally, our patient group included only veterans with combat-related PTSD and may not generalize to civilian populations or other types of PTSD, such as motor vehicle accident victims. Finally, our study did not include happy expression faces, which means we do not know what role PTSD may play, in this experiment, on positive expressions. Since our research question focused on the differences between two negative expressions, and because neutral was an important baseline facial expression comparison, we chose not to include happy faces. Using four or more expressions could create difficulties in learning and recall of key press responses and may have changed the contextual dynamic of the comparison of angry and fearful faces. Future studies could examine the role of the balance of categories of emotional faces with, for example, a series of smaller experiments comparing two, three and four emotions at a time. Similarly, a mixed block format (different target valences) versus a blocked format (same target valence consecutively) may also play a role in the outcome, a topic we examined in an emotional Stroop task (Ashley and Swick, 2009), particularly when looking at affective disorders, like PTSD (Ashley et al., 2013). Additionally, including conditions without flanking faces (target only), with scrambled faces, with color versus grayscale faces, etc., could also contribute to the understanding of the relative roles of valence and context in this task.

## Conclusion

Our comparison of PTSD patients and military controls on an emotional face flanker task using angry, fearful and neutral expression faces showed that despite both groups performing nearly identically on fearful and neutral face arrays, they may differ on angry target faces: PTSD patients responded significantly faster to congruent angry faces then military controls. This RT facilitation effect with angry, but not fearful faces, also correlated positively with a self-report measure of PTSD symptoms. These results suggest that PTSD patients may be more vigilant for, or primed to respond to, the appearance of angry faces, relative to fearful. This important topic deserves further study, especially in military populations.

## Author Contributions

VA and DS designed the experiments. VA collected and analyzed the data and drafted initial manuscript versions. VA and DS read and approved the final manuscript.

## Conflict of Interest Statement

The authors declare that the research was conducted in the absence of any commercial or financial relationships that could be construed as a potential conflict of interest.
